# Anatomical, Pathological, and Therapeutical Researches on Phthisis

**Published:** 1843-10

**Authors:** 


### Art. XIII.

*Recherches Anatomiques, Pathologiques et Thèrapeutiques sur la Phthisie.* Par P. C. A. Louis, Médecin de l'Hôtel-Dieu, &c. Deuxième Edition.—*Paris*, 1843. 8vo, pp. 688.

*Anatomical, Pathological, and Therapeutical Researches on Phthisis.* By P. C. A. Louis, Physician to the Hôtel-Dieu, &c. &c. Second Edition.—*Paris*, 1843.

The reputation attached to M. Louis' name, through the first edition of his work on Phthisis in the Adult, is likely to be very materially increased by the second version which now lies before us. The additions, and these are numerous, are invariably of that soundly philosophic character which characterizes all the productions of their author; and the full consideration of the more directly practical branches of the subject discoverable in this edition must win him the unreserved suffrages of those who, while they acknowleged his merits in the pursuit of pure science, questioned his capacity for its practical application. We shall endeavour in the following pages to make the reader acquainted with the prominent features of the work, while we at the same time describe, as completely as our space will permit, some of the chief phenomena of the all-important malady it investigates.

i. Morbid Anatomy. That the symptoms of pulmonary consumption arise solely as the result of the deposition of a certain adventitious product, called tubercle, in the lungs, is now matter of universal conviction. And if the *direct* researches of various observers of phthisis, and the general result of these researches—that individuals have never been known to die phthisical without the existence of that product being established after death—might some years since have been demurred to by cavillers on the ground that the train of functional disturbances attending the growth of cancer in the lungs, had not been made out with precision, even this source of objection is at the present hour removed. The symptomatology of pulmonary cancer has been ascertained with very considerable accuracy, and the differences between its course and that of phthisis placed in sufficiently strong relief.

Tubercle in the lung then constitutes the anatomical character of phthisis. The term is a bad one; it leads to the idea that form is an, or indeed the essential point in the physical aspect of the product—an idea quite at variance with fact, as its shape is a mere accident of position, the intimate nature of the material remaining the same, whatever be the outline—rounded, flattened, branched. &c.—which circumstances may have impressed on it. It is more correct then to speak of tuberculous matter than of tubercle; and this matter when fully developed is opaque, yellowish, firm, yet friable and of little tenacity, caseiform, occasionally somewhat unctuous to the feel, inelastic, collected into masses varying in size from that of a pin's head to a pigeon's egg, without smell, of uniform aspect all over its surface, unmarked by vessels, insoluble in water, and if mixed with this quickly subsiding to the bottom. When these characters exist, tubercle may always be recognized; if some among them be so modified by circumstances as to make the nature of the product on first sight doubtful, closer examination will commonly succeed in removing the difficulty.

But in the great majority of cases tuberculous matter as just described is found associated in the lungs with certain small bodies not much larger than a millet-seed or very young pea, of shining aspect, lilac or slightly pinkish colour, rounded or angular form, and considerable firmness: these are the semi-transparent gray granulations of many writers. So frequent is the association that tubercles were only found in two cases by M. Louis without gray granulations; and the latter without the former in five. Laennec regarded these granulations as the early condition of the yellow tuberculous matter; a doctrine supported by Louis on the following grounds. 1. Like tubercles the gray granulations are larger and more numerous at the summit than at the base, and limited to the summit, if they are not scattered over the lung generally. 2. At a certain period they present a yellowish point in their centre, which is larger in proportion as it is nearer the apex of the lung. 3. The two products are, as already mentioned, almost invariably associated. 4. The gray matter occurs in infiltrated masses also, and then too presents yellow tuberculous points in its interior. 5. In some instances gray matter undergoing the yellow transformation, is found in other organs besides the lungs. We believe that these arguments (the fourth excepted which, as will be by and bye seen, possibly involves an error,) have not been satisfactorily set aside by any of the advocates of the mutual independence of the gray granulation and yellow tubercle. And the notion of Dr. Carswell especially, that the gray granulation in the lungs is tuberculous mixed with mucous matter, which two materials subsequently separate from each other,—the former accumulating towards the centre, the latter at the periphery of the mass,—appears to us destitute of all probability.

This question, as well as all others appertaining to the intimate history of tubercle, would appear perhaps to be capable of easy elucidation with the assistance of the microscope. But the contrary is unfortunately the truth; at least the most diligent micrographers give accounts so discordant upon this point, not only in seeming but sometimes in reality, as to justify us in concluding that the study of tuberculous matter is one beset with no ordinary difficulties. Kuhn, Gluge, Vogel, Henle, Gueterbock, Wood, Gruby have laboured in this field without succeeding in establishing the micrology of tubercle, or satisfactorily aiding the pathologist in his inquiry into its mode of origin; and amid the conflicting statements tendered, perhaps that of the essential characteristic being a finely granular substance is the only one that can be received without hesitation. The granules composing this, dark coloured, closely packed, and of minute size, measure from 1-12000th to 1-15000th of an inch in diameter. This is the condition of tuberculous matter which we just referred to as clearing up any doubts that might be entertained of its nature. M. Louis gives certain results, obtained by a M. Lebert, from the microscopical investigation of pus, cancer, and tubercle; but as these results do little more than prove their author to be in the noviciate of his studies of minute structure, it is certainly needless to detail them.

It will not be necessary for us to follow closely the anatomical history of tubercle of the lung in all its bearings; more especially as our space may be more profitably employed in the extended consideration of some particular and debated points. One of these is the nature of the homogeneous, grayish, "semi-transparent," shining, firm mass, encountered in many cases around tuberculous cavities, and more rarely independently of these. M. Louis retains the precise views respecting this appearance formerly promulgated by him,—he regards it in part as the result of infiltration of semi-transparent tubercle (of the material of the gray granulation) through the pulmonary tissue; in part as the produce of chronic inflammation. We confess we can see no very urgent motive for considering that the appearances referred to are ever other than the results of chronic inflammation: the fact of its frequently presenting gray granulations or yellow tubercles on its surface proves nothing, for there is assuredly no reason (to state the matter in the least dogmatical manner) why those formations should not occur in parts infiltrated with the product of simple chronic inflammation. The characters of the pulmonary tissue thus affected are certainly not obviously and materially different from those observed in simple chronic pneumonia occurring without tuberculous disease. The author doubts the identity of the gelatiniform tuberculous infiltration of Laennec with the gray semi-transparent matter; no doubt correctly,—Andral long since contested the accuracy of Laennec's views upon this point.

M. Louis makes no alteration in the statements promulgated in the former edition respecting the greater proneness of the left than the right lung to suffer from tuberculous disease. Numerous facts have nevertheless been collected of late years showing that some doubt may be entertained of his perfect correctness in this matter; fuller allusion to these will be found in a former volume (Brit. and For. Med. Rev., Vol. IX. p. 334,) where we also made it evident that M. Louis' own cases do not so obviously support his opinion as he himself appears to imagine.

The duration of the several anatomical stages of the malady is approximatively estimated by M. Louis; but the difficulty of its determination has not appeared less to him than it has done to others. The time necessary for the growth of the gray granulation to the size of a pea is, for example, impossible to determine in general terms; certain cases of acute phthisis appear to show that it may attain this bulk in the course of two or three weeks; while, on the other hand, the author has met several instances in which persistent cough and occasional hemoptysis had existed for years, and yet gray granulations of the size mentioned, or even smaller, were the only pulmonary lesion discovered. Nor is the period of softening less subject to vary,—in some cases occurring from the twentieth to the fortieth day, in others after a much greater lapse of time. The end of the third or beginning of the fourth month are the earliest periods at which M. Louis has established the existence of empty cavities.

The distinction of simply purulent from tubercular cavities, though commonly easy and justified by a multitude of conditions familiar to the pathological anatomist, is in some instances a matter of real difficulty. M. Louis' third case exemplifies this. Here was a young woman who had coughed for seven months previously to her admission, and dying, seventeen days after, presented at the summit of the right lung an excavation of moderate size, partly filled with a turbid, greenish fluid, and containing a piece of lung perfectly free from all connexion with the rest of the organ. The cavity was lined with a false membrane of moderate consistence, a quarter of a line thick, and in contact with *healthy* pulmonary parenchyma. There were neither tubercles or gray granulations in the lungs; nor ulcerations in the larynx, trachea, or intestines. M. Louis nevertheless regards the excavation as tuberculous, because the purulent matter it contained and the false membrane lining it were perfectly similar to those of acknowledged tuberculous cavities, and because "one of the cervical glands was in part tuberculous." The last argument the author regards as not the least conclusive of the three : in this we coincide, if the matter was *really* tuberculous, but we cannot grant that, even with its aid, the tuberculous character of the cavity is fully made out. Its anatomical characters differed in no wise from those of an abscess; and the existence of a detached fragment of lung in its interior, a circumstance without a parallel, as far as we are aware, in the history of undoubted phthisical cavities, is not so uncommon a condition in simple purulent collections. The existence of the excavation at the apex might, on first consideration, appear to support the idea of the tuberculous nature of the lesion, but the fact is that abscess is more common in the superior than the inferior lobes. Thus, Grisolle (De la Pneumonie, p. 53,) found that of 19 pulmonary abscesses 9 were in the upper lobe, 5 in the inferior, 1 in the middle; and in four instances there were several purulent collections in different situations. Nor do the commemorative symptoms bear out M. Louis' view. The patient was admitted fifteen days after delivery, which had been natural and at the full time ; but at the close of pregnancy, rigors followed by heat and sweating had supervened and ceased after parturition. Hemoptysis had never occurred.

The great additional experience acquired by the author since the publication of his first edition has not enabled him to alter the statement there made, of his never having observed in the midst of *healthy* pulmonary tissue cavities communicating with the bronchi and lined, like *old* tuberculous cavities, with a grayish, semi-cartilaginous, and incompletely opaque false membrane. He admits, however, that such appearances have actually been observed by Laennec and many of his successors, and considers the notion of their signifying the cure of phthisis confirmed by the circumstances of the case to which we have just made brief allusion. But we have shown the deficiency of proof of the tuberculous nature of the disease in that instance; and must observe that the experience of Louis, as above stated, goes to support, in the very strongest manner, the view which we believe a deep study of all the alleged cases of production of innocuous cavities from tuberculous destruction of the lung (without the presence of tuberculous disease in other forms,) will amply justify, namely,—that these cases are nothing more than examples of excavation following the evacuation of simple inflammatory collections of pus.

Nor has M. Louis during his researches, pursued through a period of twenty-five years, met with a single example of those masses of condensed cellular tissue in which bronchial tubes more or less dilated are alleged to terminate, and which Laennec regarded as cicatrices of tuberculous cavities. He is far, however, from denying either the reality of their occurrence or the justness of Laennec's views of their curative signification. A mode of cure, admitting it to be such, which does not exhibit itself once in such an enormous number of cases as must have presented themselves to M. Louis during so lengthened a period, can, practically speaking, be little more than valueless.

The depressions frequently observed at the upper part of the lungs, along with corrugation of the surface around, appear to M. Louis to be unconnected with any determinate lesion ; like all other pathologists he has seen them on lungs the substance of which was perfectly healthy.

The only addition we discover to the details descriptive of the bronchi, consists in the account of a "tuberculous false membrane," found in a single instance in the interior of one of the larger bronchi, and lining the tubes from place to place to the very periphery of the lung. The anatomical characters of this production are not given with much precision.

The readers of Dr. Carswell's fasciculi are well acquainted with his description and accompanying figure of atrophy of one lung, produced by the presence of tuberculized bronchial glands on its main bronchus. This condition observed in the monkey has not yet been noticed in the human subject. Dr. Carswell ascribes the atrophy to deficient entry of air into the vesicles, and the consequent functional inactivity of the organ. Possibly the position of the bronchial arteries is in that animal such as to expose them more readily than in the human subject to extraneous pressure; if so the atrophous shrinking of the lung may have, in some measure at least, depended upon this cause. M. Louis in commenting on the case, (which was originally seen, and has since been described by Reynaud,) notices the possibility of a similar condition of things arising in man, and points out the almost inevitable error which would be incurred in its diagnosis during life.

M. Louis conceives that the most important addition to the morbid anatomy of tuberculous disease, made since the publication of his former edition, is comprised in the results obtained by Schroeder Van der Kolk, and Guillot, respecting the vascular condition of the lungs. The Dutch author has in this matter all the merit due to its original investigator; but as the observations of M. Guillot are the more complete, these we shall condense. It is ascertainable that the branches of the pulmonary artery stop or cease to be permeable at a distance of three, four, or five millimeters from tubercles or gray granulations ; the length of vessel impermeable increases with the augmentation of the tuberculous masses, so that when these are considerable, or when they have given place to cavities, a sort of investment, two centimeters thick, may be found around them, presenting not a single ramification of the pulmonary artery. By injection and microscopical examination, it is further discoverable that this total absence of vascularity is only temporary; after a time, red lines, tapering off at either end, and in their widest part equalling a millimeter in diameter become discernible. At first these vessels are perfectly isolated, but in process of time communicate with the bronchial arteries, or with those of the walls of the thorax. The latter communication is effected by means of new vessels developed in the pleural false membrane, (the particular discovery of Van der Kolk.) The amount of new vascularization effected in this way increases greatly with the progress of tuberculous destruction ; the rete spreads eventually, it may be, through a great part of the affected lung, and replaces the system of the pulmonary artery which has ceased to be discoverable.

An enquiry naturally presents itself, as to the influence on oxygenation exercised by this novel condition of supplementary vessels. It is in truth aortic blood, that by means of the bronchial arteries and new system of vessels spreads through the lungs; and M. Guillot has ascertained that this blood must return to the heart by the bronchial, pulmonary, and azygos veins, as he ascertained that the substance of injection thrown in by the aorta is found in these veins. Now this condition of circulation is one that manifestly cannot subsist without materially altering the blood of phthisical subjects, and thereby affecting their organization generally. The main result in respect of function may be expressed thus,—that in proportion as tuberculization advances, the lungs acquire increasing capacity for arterial, and loose it for venous blood.

The researches of M. Guillot upon the elementary and primary seat of tuberculous matter, lead him to follow the views of Dr. Carswell, and place this in the bronchi as soon as the accumulation is sufficiently great to be distinctly and satisfactorily examined. In the earlier stages he thinks it a just inference from what is thus ascertained in more advanced ones, that the ultimate terminations of the bronchi are the seat of the change. M. Louis esteems the attempt to limit the lesion to a particular texture, a labour which can lead to no useful result,—an idea, as it appears to us, of most questionable wisdom.

The author invokes the authority of Dr. Carswell, (as employed by M. Valleix in an essay on the subject,) in support of his own objections to the notion of M. C. Baron, that tubercle originates in modification of extravasated blood. He observes that the researches of the English pathologist have placed beyond a doubt the existence of the gray semi-transparent granulation in perfectly rudimentary tubercles. M. Valleix has altogether mistaken and led M. Louis into a mistake respecting the opinions of Dr. Carswell. He is in truth one of those who most strenuously resist the notion of a necessary connexion between the gray granulation and yellow tubercle; and regards it in the lung as mere bronchial mucous, separated by filtration from the tuberculous matter, in company with which it has been accidentally secreted.

Pneumonia is far from uncommonly the ultimate phenomenon of phthisis; but there is nothing characteristic in this, the proportion of cases in which it occurs not being materially greater than in other chronic diseases taken indiscriminately. In other words, tuberculous disease does not *per se* exercise any special influence in generating the *final* pneumonia met with in a certain number of cases of phthisis,—hence it is rather a phenomenon evidential of the *chronic* than of the *tuberculous* character of the malady. Not so, however, of the pneumonia which frequently occurs as an intercurrent affection in phthisis; here the tuberculous influence is manifest.

The two pleuritic lesions which the researches of M. Louis mark as peculiar to phthisis are, " the sort of cartilaginous cap covering the apex of the lung when excavated," and tubercles developed in the false membranes of the pleura. With regard to the latter point, M. Andral states that " in *almost* all the cases where he has met with tubercles in the pleura, he has also detected them in the lungs;" from which, of course, the reader is to infer that he has actually seen some cases of tuberculization limited to the pleura. Three years ago, (Brit. and For. Med. Rev., Vol. IX. p. 325, April, 1840,) we drew attention to a statement of similar purport by M. Fournet, and allowed our own doubts as to the really tuberculous nature of the alleged pleural tuberculization to be inferred. We have, since that period, met with some instances of the condition by which MM. Andral and Fournet have been betrayed into the bootless contradiction of one of the most important practical laws of pathology, and easily ascertained that the so-called tubercles were nothing more than common coagulable lymph, deposited in an irregularly granular form. M. Louis upon other grounds similarly denies the reality of tuberculization limited to the pleura.

The section devoted to the morbid anatomy of the larynx, trachea, and epiglottis is enriched by the analysis of a very large number of examinations of those organs made by the author since 1825. We shall throw these as far as possible into a tabular form, for the purpose of economising space.

| No. of cases. | | Ulcerations in Trachea. | |
|---|---|---|---|
| 190 | 80 Females | 21 | = 1/4 |
| | 110 Males | 55 | = 1/2 |
| | | 76 | = 1/3 and upwards. |

| No. of cases. | | Ulcerations in the Larynx. | |
|---|---|---|---|
| 193 | 80 Females | 19 | = 1/4 about. |
| | 113 Males | 44 | = 1/3 " |
| | | 63 | = 1/3 " |

| No. of cases. | | Ulcerations in the Epiglottis. | |
|---|---|---|---|
| 1 | 47 Females | 8 | = 1/6 |
| | 87 Males | 27 | = 1/3 about |
| | | 35 | = 1/4 |

| No. of cases. | | Ulcerations in the Bronchi. | |
|---|---|---|---|
| 49 | 19 Females | 5 | = 1/4 |
| | 30 Males | 17 | = more than 1/2 |
| | | 22 | = 1/2 nearly. |

These facts show the remarkable degree of frequency of ulcerations of the air-passages generally in this disease, the gradual increasing frequency with which they are discovered, as the parts examined are more and more deeply seated, and their comparative rarity in females. We are utterly at a loss to account for the influence of sex signified by the last-named peculiarity; that ulceration of the tubes should be more common in the immediate neighbourhood of cavities than higher up the air-passages, is nothing more than may be readily understood from the more protracted sojourn of the sputa in the former than in the latter situation,—at least, if we admit the justness of M. Louis' views as to the causation of ulcers by the irritation of these sputa. And there is, in truth, every motive for accepting those views as explanatory of the *general* fact of ulceration; however, M. Louis himself points out that ulcerations *may* exist in rare instances in the mucous membrane of the air-passages, without the presence of cavities. What their cause may be under such exceptional circumstances, it is not easy to determine, for the laborious author has never in a single instance detected tubercles in the mucous membrane of the upper air-passages.

The ulcerations we have been now referring to, though never directly of tuberculous character, were nevertheless set down by M. Louis as peculiar to phthisis, because never met with except in subjects labouring under that disease: syphilitic ulcers are of course excluded from this statement. In upwards of 500 non-tuberculous subjects dying of various chronic diseases examined since the publication of the former edition, M. Louis did not find one example of laryngeal ulceration; it is therefore but just to conclude, (and the experience of every physician whose practice leads him to study these maladies seriously, will, we believe, harmonize with the conclusion,) that chronic laryngitis with ulceration, is but a part, a secondary lesion, of phthisis.

We discover no new statements in the chapter relating to the state of the heart; the author simply refers to the corroboration of his former results, respecting the diminished size of that organ in phthisis, derived from the minute investigations of Bizot. (Vide Brit. and For. Med. Rev., Vol. VI. p. 43.)

The pharynx and œsophagus were almost always in the natural state; in 120 subjects the former was ulcerated in but four cases,—the latter in six. The œsophagus was pretty frequently coated with pultaceous exudation,—a condition not productive of any particular symptom.

The size and position of the stomach undergo modification in phthisis with moderate frequency. Of 96 subjects in whom these points were carefully examined, 9 presented enlargement of the organ to double or treble its natural size, and in 6 of these cases the great curvature was on a level with the crista of the ileum. In all these instances the liver was enlarged and depressed also. Now in 230 subjects dying of various other (acute or chronic) diseases, these peculiarities were only encountered twice: M. Louis, then, regards them, when so highly marked as we have described, as almost proper to phthisis and probably induced by the constant efforts of cough.

The opinions formerly held by the author on the subject of "softening with attenuation of the mucous membrane of the stomach," were materially, if not completely modified, as the readers of this Journal are aware, in the last edition of his treatise on typhoid fever, (vid. Brit. and For. Med. Rev., Vol. XII. p. 25,) the essentially chemical and cadaveric nature of the appearances was then admitted, although it was very fairly urged that there are some circumstances connected with the conditions under which solution takes place, as yet undiscovered. The other kinds of anatomical change found in the *mucous membrane* in 96 cases carefully examined were as follows:

| | |
|---|---|
| Attenuation . . . . . . | 19 |
| Redness, and sometimes thickening, mammillation and softening anteriorly | 8 |
| Softened, and of obscure red colour in the fundus . . | 17 |
| Mammillated, grayish, sometimes reddish, thickened, &c. . . | 19 |
| Ulcerated, without other lesion . . . . . | 2 |
| Softened, without alteration of colour or thickness . . . | 4 |
| Redness more or less florid over entire surface, without alteration in point of thickness or consistence . . . . . | 6 |
| Viscid fluid underneath it . . . . . . | 1 |
| Presenting cicatrices . . . . . . | 1 |
| | 77 |

These lesions are none of them peculiar to phthisis; the author found them in one half of the subjects he examined, dying of all other chronic diseases indiscriminately. However, as appears from the above table, they are much more frequent in phthisis, existing in four fifths of its victims. The presence of tuberculous matter either in the substance of, or underneath the mucous membrane here, is singularly rare: M. Louis has examined 400 stomachs without once detecting it, and M. Andral encountered it but twice in several hundred cases. This circumstance is dwelt upon by the author as indicative of the non-dependence of tuberculization on inflammation, for, as we have just seen, the stomach is very frequently the seat of the latter morbid state.

The author has found the proportion of cases of duodenal ulceration, (9 out of 60,) higher than that formerly announced. In one case he observed a few small sub-mucous tubercles. He makes no remark regarding the state of Brunner's glands; organs which, according to the statements of a recent German writer, are sometimes distinctly tuberculized.

The condition of the mucous membrane of the small intestine in its various changes, from the first deposition of tubercle in its systems of agminated glands to perforation of the bowel, is detailed with that graphic precision which constitutes one of the most remarkable features of all the author's writings. There are no novelties in the details, however, with a single exception to be immediately considered, and we therefore content ourselves with strongly recommending their minute and complete study. The point we desire to refer to here is the occasional occurrence of cicatrization in some one or more of the ulcerations of the small intestines. On first thought, such cicatrization might appear a fortunate circumstance for the individual in whom it occurred, but a little reflection upon the anatomical constitution of cicatrices of mucous membranes, and their influence on surrounding tissues, will lead us to doubt even the innocuousness of a cure of the kind. In truth the plastic exudation forming the staple material of cicatrix, is endowed with a property of contraction, the tendency of which must be to produce diminution of the caliber of the gut. And the observed fact is occasionally so; such contraction has arisen in not a few cases on record, and in one added to the number by M. Louis, the symptoms of obstructed bowel were so marked, and so pertinacious, (eventually they proved the cause of death,) that they diverted attention from the state of the lungs, which was not inquired into,—the pulmonary disturbances being masked by the intestinal. Here then we have a new—at least a newly-known—mode of death by tuberculous disease; and by the fact of its possibility, we are led to infer that it is better for phthisical subjects *not* to have their intestinal ulcerations cured, than run the risk of that cure causing their destruction. There is, of course, only a risk, not a certainty,—for contraction is not an unfailing attribute of intestinal cicatrization. The merit, however, (we have much satisfaction in adding,) of pointing out the danger of cicatrization of ulcers in the small intestine, does not belong to the French school; Dr. Carswell, years ago, observed and described in one of his Fasciculi this very condition, productive of the fatal issue of a case of ulcerative continued fever about twelve months after imperfect convalescence.

It is to be observed that ulceration of the small intestine is almost peculiar among chronic diseases to phthisis; at least 85 subjects dying of all other chronic affections presented but 6 examples of such ulceration, and in three of these six there were tubercles in the lungs.

Intestinal ulceration is frequently independent, originally, of inflammation. The development of the small tuberculous masses cannot itself be ascribed to inflammation, because the mucous membrane adjoining them is unaffected so long as they remain unsoftened.

M. Andral inquires, in the last edition of the Clinique Médicale, how the very common belief in the frequent coexistence of fistula in ano with phthisis has arisen,—he himself having only ascertained its existence once in 800 tuberculous subjects? M. Louis states that he has not been more successful than his countryman in the search after fistula, but gives no numerical statement of his experience. This appears the more remarkable as the frequency of ulceration in the large intestine is so great that M. Louis found this change more or less extensively developed in 88 cases out of 108 cases. We have long been satisfied, from the repeated interrogation of tuberculous subjects regarding the state of their anus, that the common opinion is absurdly exaggerated, and that probably fistula in ano is not more common among them than in all other kinds of unhealthy individuals taken indiscriminately; but, on the other hand, we are firmly persuaded that, in this country at least, fistula is more common than M. Andral's experience would lead us to suppose. We have ourselves met with at least 4 cases in about 200 tuberculous subjects, observed in hospital within the last twelve months; and we should think that the proportion witnessed by us, in private practice (although we speak without positive records,) must be considerably greater than this.

The chapter relative to the lymphatic glands is very considerably enlarged in the present edition. The frequency of tuberculization of these glands in the different regions of the body is shown by the subjoined table:

| Lymphatic Glands. | No. of cases examined. | | Tuberculous in |
|---|---|---|---|
| Cervical . . | 80 | ... | 8 = $\frac{1}{10}$ |
| Bronchial . . . | 70 | ... | $\frac{1}{2}$* |
| Mesenteric . . | 102 | ... | 23 = $\frac{1}{4}$ |
| Meso-cecal and meso-colic | " a little less frequently than the mesenteric." | | |
| Lumbar . . | 60 | ... | 5 = $\frac{1}{12}$ |
| Axillary . . . | | ... | 1 |

Tuberculous enlargement of the cervical glands does not commonly induce notable symptoms; in one case, observed in the infant by M. Tonnelé, the vena cava superior was pressed upon by a tumour of this kind, and a stasis of the blood, extending to the jugular veins and sinuses of the brain, entailed.

* There is a typographical error in the original, either in the number of cases or the proportional frequency; the context renders it much the more probable if not actually certain that it is in the former, we therefore give the latter. It is to be observed, with respect to the state of the bronchial glands, (and, indeed, the circumstance must invariably be borne in mind throughout this article,) that the researches of M. Louis refer to the disease as it exists in subjects aged upwards of 15—younger individuals being excluded from the hospitals in which he observed. Tuberculization of the bronchial glands is in infancy more frequent even than that of the lungs.

Although bronchial tuberculization is much less common in the adult than infant, yet it is a sufficiently interesting condition; and one of the peculiarities attending its progress in infancy have been well made out by MM. Rilliet and Barthez. The bronchial glands are generally invested by a cyst with thin walls, to which the tuberculous matter closely adheres. At a certain stage of advancement the cysts contract adhesions with adjoining parts, much more frequently with the bronchi than with the pulmonary substance or vessels. After this union takes place the softening of the tuberculous matter generally follows, and subsequently the establishment of communication between the tuberculous cyst and the bronchi. At this period the cyst becomes lined with a red false membrane, intimately united with the mucous coat of the bronchi. In 26 cases such communication of tuberculized glands and the air-passages was discovered 18 times; 12 times on the right side, 5 on the left. Pneumo-thorax (without corresponding symptoms, however,) followed in one case of perforation of the cyst. M. Louis appears to regard these observations as completely new; the truth is, that Dr. Carswell, years ago, figured a case (Fasciculus, Tubercle,) illustrating the whole process of evacuation of altered tuberculous matter through the trachea and bronchus, from the interior of the tuberculized bronchial glands; and exhibiting the condition of cicatrized trachea which sometimes follows, and which, from the details of our author, appears not to have attracted the attention of the French observers. M. Berton once saw perforations of the pulmonary artery similarly produced.

Those who are acquainted with the arguments used by Dr. Carswell in support of his notion of the independence of the gray granulation in the lung and yellow tubercle, are aware that he (and others who adopt his views) refers to the total and invariable absence of the gray semi-transparent matter in the lymphatic glands as a point of very material importance in their favour. In all M. Louis' cases of tuberculous disease of these glands, it would appear that but two instances occurred of such development; but these are enough to show that the position of Dr. Carswell is untenable. And we are persuaded that the relative frequency of gray matter in the mesenteric glands is sometimes greater than the statements of M. Louis would signify: we have detected it in four or at least three (for one instance might be cavilled against) cases out of some twenty post-mortem examinations, in which the state of those glands was closely examined.

Tuberculization of the lymphatic glands never occurs, according to M. Louis (and we believe him of *true* tuberculization,) after the age of fifteen, unless in phthisical subjects.[a]

The peculiar alteration of the liver which M. Louis' previous volume contributed especially to make known, under the name of "fatty transformation," is extremely frequent in subjects dying of phthisis in France. Forty out of 120 individuals presented this condition of liver. The organ, when thus affected, is of faded-leaf tint, enlarged, but unaltered in general shape; of diminished specific gravity, and capable of greasing bodies brought in contact with it. The lesion is four times as frequent in females as in males, and appears to be almost peculiar to phthisical persons. The transformation may be very rapidly effected, according to M. Louis, because he has discovered it in cases of phthisis which had run their course in fifty days; herein the author assumes its dependence upon and the impossibility of its existing without phthisis, as granted. It produces no symptoms of appreciable character, and the author regards enlargement of the liver (as discoverable by protrusion of the organ beyond the free border of the ribs) as the only circumstance capable of leading to its diagnosis during life. But this protrusion is insufficient in itself to prove the fact of enlargement; it may announce depression only. And it is to be observed that fatty liver is much more rare in this country than in France,—a circumstance which we are at a loss to explain. Indeed, the whole etiology of the condition is infinitely obscure.

Four subjects only had biliary calculi; in one of these cases the usual symptoms had existed during life, in the three others they gave rise to no inconvenience.

The kidneys were perfectly natural in respect of consistence, colour, and size in three fourths of the cases. In 16 out of 90 cases they were slightly redder than natural; in 3, of much greater consistence than usual; in 4 subjects they contained small serous cysts; in 3 instances were the seat of tuberculous matter, speading in one of these into the corresponding ureter. This unity of tubercular disease in the kidney is confirmed by the author's subsequent experience, for 80 post-mortem examinations supplied but two examples of it. Rayer's statement would lead us to suppose the condition of more frequent occurrence than these results of M. Louis warrant. In a single instance among 214 post-mortem examinations the author found the kidneys in a state of fatty transformation. And in nearly 500 subjects dying of all other diseases except phthisis, not a single example of tuberculous or fatty affection of the kidney occurred.

The penis presented nothing remarkable in the few instances in which it was submitted to examination : in 40 subjects in which the prostate, vesiculæ seminales, and vasa deferentia were closely dissected, these parts were all three found tuberculous once and the prostate alone twice. M. Louis has never seen the urethra tuberculous; Rayer, however, quotes two cases of the kind.

Morbid change is rare in the genital organs of female tuberculous subjects; the size of the uterus is, however, commonly below the healthy standard. As in persons dying of other affections, the author found uterine polypi in a certain number of cases (we regret he has not specified the proportion, for no slight difference of statement exists in different works upon the point.) In one case referred to in the former edition, the internal surface of the body and neck of the uterus was invested with a stratum of tuberculous matter, and the tissue of the organ, otherwise healthy, studded with minute yellow tubercles. Among more than 200 uteri of phthisical women examined by the author since 1825, three examples only of tuberculous disease were discovered. The fallopian tubes were, in a case described by Reynaud, crammed with the morbid matter. Ulceration of the vagina appears only to occur when the tuberculous substance escapes from the organ and is found along that canal,— a mode of ulceration analogous to that already described in the trachea. These various conditions have been delineated by Dr. Carswell.

The importance, both pathologically and practically, of the chronic peritonitis of phthisis is well established by the chapter on lesions of the peritoneum. In the cases where that affection existed the other organs of the body were, comparatively speaking,—this is true even of the lungs,—in an incipient state of disease; hence the peritonitis evidently had a considerable share in hastening the fatal event. This view is confirmed by the circumstance that in several of these cases the disease ran its course very rapidly,—in one instance in forty-four days. The development of this peritonitis is not fairly attributable either to the influence of sub-peritoneal tubercles or to that of intestinal ulcerations, for in several cases these lesions were altogether wanting, or were present to a very limited amount. The author concludes the action of some special cause (as in the case of various of the other secondary lesions of phthisis,) aided by the violence and constancy of febrile action. Whatever the circumstances are which favour the generation of chronic tuberculous peritonitis, they influence the pleura also : for of 13 cases of the former, 4 were attended with tuberculous pleuritis. It is tolerably well known that, in his former edition, M. Louis maintained chronic tuberculous peritonitis to be peculiar to phthisis, as he had never seen the state in non-consumptive subjects. All his subsequent experience corroborates the announcement then made ; among upwards of 300 individuals dying of other chronic diseases besides phthisis, not one presented the anatomical characters of tuberculous peritonitis.

Although the functions of the brain are almost always unaltered in phthisis even to the last hours of life, yet some lesion or other of that viscus or its membranes is discoverable after death in a considerable number of cases. The cerebral arachnoid was frequently thickened partially, and contained non-tuberculous granulations in greater or less numbers, especially in the neighbourhood of the falx. It was invested in two cases by a soft yellowish false membrane. The sub-arachnoid cellular tissue was infiltrated with serosity, and the lateral ventricles distended with the same fluid in three fourths of the cases. In a certain number of instances the sub-arachnoid tissue contained (especially in the fissure of Sylvius) a variable quantity of semi-transparent or tuberculous granulations. These granulations, which form the anatomical character of tuberculous meningitis in infancy, (at which period of life they are sufficiently common) have scarcely been submitted to the fitting study in the adult. The inquiries of M. Lediberder have merely excited interest in the matter without establishing the facts connected with it. In a seventh part of M. Louis' cases the brain was more or less injected ; in the twentieth part of them, softened throughout its entire mass, once very remarkably so. It seldom contained tubercles.

The general result of all the author's experience since 1825 is to confirm the remarkable and well-known statement then made by him to the effect that after the age of fifteen tuberculous matter never presents itself in any tissue or organ unless it exists also in the lungs. To the general law he admits he has observed one, and refers to the existence of a second, exception ; but the exterior paucity of such exceptional occurrences places in most prominent relief the importance of the author's discovery. And we may add that having become acquainted with the law in 1833, we have not ourselves either seen or heard of a single authentic case falsifying its provisions.

II. Symptomatology, &c. Too much stress can scarcely be laid upon the importance of acquiring an accurate notion of the mean duration of phthisis, and yet no task of greater difficulty probably can be assigned the physician. He who has practically and, at the same time, conscientiously endeavoured to ascertain the period of origin of the disease in a considerable number of cases, will fully bear us out, we doubt not, in this view of the difficulty of the attempt. We are indeed persuaded from our own experience that, especially among patients of the class frequenting hospitals, it is not seldom a matter of impossibility to satisfy one's self as to the accuracy of the averments made respecting the outset of disease. And it is for this reason that we are gratified to find M. Louis speaking of his results as drawn from cases in which the duration of the disease was ascertained "with all the precision possible," and not dogmatically presenting them as positively and absolutely accurate. It is besides obvious that the *real* duration of the malady from the first hour of anatomical change can never—no more than in the instance of cancer, for example—be accurately made out; all we can hope to arrive at by the rejection of doubtful cases and the collection of a vast number of facts, is the mean duration of the disease after it has given symptomatic evidence of its existence,—in other words, its average *apparent* duration. The latter is fortunately that which the practical observer has the closest interest in the establishment of.

In the former edition of his work M. Louis gave the duration of 114 cases; in the present he adds that of 193 more. We shall place together these two series of results:

| Duration. | No. of cases. | Duration. | No. of cases. |
|---|---|---|---|
| 22 days | 1 | 10½ months | 1 |
| 24 " | 2 | 11 " | 10 |
| 30 " | 1 | 12 " | 13 |
| 35 " | 2 | 12½ " | 3 |
| 39 " | 2 | 13 " | 2 |
| 40 " | 1 | 13½ " | 2 |
| 45 " | 1 | 14 " | 9 |
| 50 " | 2 | 14½ " | 2 |
| 52 " | 1 | 15 to 16 | 10 |
| 60 " | 1 | 17 to 18 | 7 |
| 70 " | 1 | 19 " | 5 |
| 75 " | 1 | 20 " | 4 |
| 80 " | 1 | 21 " | 2 |
| 81 " | 1 | 23 " | 3 |
| 84 " | 1 | 24 " | 18 |
| 3 months | 7 | 28 " | 1 |
| 3½ " | 5 | 30 " | 3 |
| 4 " | 17 | 36 " | 9 |
| 4½ " | 2 | 39 " | 1 |
| 5 " | 19 | 48 " | 11 |
| 5½ " | 4 | 60 " | 6 |
| 6 " | 25 | 66 " | 1 |
| 6½ " | 2 | 72 " | 4 |
| 7 " | 16 | 84 " | 1 |
| 7½ " | 6 | 90 " | 1 |
| 8 " | 17 | 10 years | 1 |
| 8½ " | 1 | 12 " | 2 |
| 9 " | 20 | 14 " | 1 |
| 9½ " | 2 | 20 " | 1 |
| 10 " | 10 | | |

Hence it follows that out of a mass of 307 patients 4 died within the first month, 14 within two months, 26 within the first three months, 98 or about one third of the whole number, within the first six months;—facts sufficiently demonstrative of the rapid progress of the disease in a large proportion of cases. In two years 264 of the total number are gone, leaving only 43 to drag on their weary existence. The author has not calculated the mean duration of the malady from his individual facts: we find it to be from seventeen to eighteen months, taking the entire mass of cases; but if the five cases in which the disease lasted ten years or upwards be omitted from the calculation, the average duration is reduced to from fourteen to fifteen months. It is to be observed that these are calculations of apparent duration, as established by means of symptoms,—the real duration is possibly *materially* greater.

The series of cases given in the present edition furnishes a lower mean than that of its predecessor; seven years and a half is the longest duration noted in the former,—all the instances of a protracted course of ten, twelve, fourteen, and twenty years are extracted from the first.

The mortality produced by phthisis, in the wards of M. Chomel, where the author's first collection of cases was taken, was really enormous, being in the proportion of one to two to the total amount of deaths. And if to the number of persons actually dying from phthisis be added that of subjects who, perishing of other affections, had nevertheless tubercles or tuberculous cavities in the lungs (forty persons appeared in this category,) we have a total mass of 163 tuberculous deaths (if the expression be admissible) out of 358 occurring in the wards of La Charité. There is too much reason to believe that the proportion would be found equally high among ourselves, had we some physician, possessing the devotion and the opportunities of M. Louis, ready to investigate the question on the same principles in the wards of our hospitals. It is obvious that the questions borne upon by such investigation are of a totally different character from those answered by the invaluable reports issued by the Registrar-general, and illustrated by Mr. Farr.

The consideration of the duration of the disease is followed by a particular description of each symptom occurring during its progress: these descriptions will furnish us from time to time with material for comment.

The author states, on the evidence of cases recorded in the work, that decumbiture on the side of the principal excavation was insupportable from the increase of cough it entailed; to such a degree that, independently of more certain means, the decumbiture of the patient will aid the observer in discovering the side at which the disease is most advanced. Generally speaking this is true, but there are other circumstances which influence the patient in choosing his posture; and where a very closely similar amount of disease has been found in both lungs we have known the patient steadily maintain one position.

The opaque, globular, striated, ragged sputum, containing no air, is justly regarded by the author as extremely significant of the disease; nevertheless he has in two instances seen this condition of expectoration independently of tuberculous affection; and in three cases of phthisis, on the other hand, the sputa remained mucous, aerated, whitish or slightly yellowish or grayish, and semi-transparent to the end.

The variation in respect of quantity of expectoration, both in different cases and in the same case, at different times is well known to be remarkable. We have ourselves observed instances in which a total suspension of expectoration persisted for several days at a time, and this without any obvious coexisting improvement in other *symptoms*,—the phthisical *signs* were of course altered. The author refers to one case, indeed, which ran to the end without the patient having spit at all; though the lungs contained large cavities. Such a fact as this appears inexplicable except on the supposition that, as is almost universally the case with children, the patient, in this instance an adult, swallowed her sputa; nevertheless this view of the case is, on the other hand, not without its difficulties. We have seen one such case; the subject of it was a boy, aged fourteen. Sir James Clark, too, states that he has met with one example of total absence of expectoration. It is important that the practitioner should be aware of the reality of such cases.

Occasionally during the second period of the disease patients void an enormous quantity of puriform-looking sputa, which cannot be ascribed to the sudden evacuation of a large mass of softened tuberculous matter. When effusion into the pleura has coexisted with the parenchymatous disease in such cases, the abundant expectoration has commonly been ascribed to escape of the pleural fluid through a perforation of the lung, but this is by no means a necessary occurrence or an invariably just explanation. The coincidence referred to, was observed by M. Louis about a year since at the Hôpital Beaujon; here the subject, who had suddenly voided an enormous quantity of puriform matter, had not presented the symptoms of perforation, nor had the level of the effusion fallen after the copious expectoration, which must inevitably have been the case had that effusion been its source. M. Louis concludes that a *sudden and temporary increase in the secretion of the bronchi and cavities* is capable of producing the occurrence in question.

The expectoration of calcareous particles from the lungs of tuberculous subjects is not by any means so common, as might be anticipated. M. Louis has never met, either in hospital or private practice, with a single example of the fact. We have ourselves, in a certain number of instances, been assured by patients, and this very circumstantially, that they had expectorated such matter, but we are little disposed to confide in accounts of the kind, and we have *seen* but one example (the specimen is before us) of the occurrence.

The general history of the hemoptysis of phthisis is perfectly well known; two or three points regarding it, however, deserve particular notice. The common belief of the frequency with which hemoptysis proves the immediate cause of death in phthisis would appear, from M. Louis' experience, to be an incorrect one; at least no such case had presented itself to him at the period his first edition was published, and out of three hundred phthisical persons since observed three only perished of hemoptysis. We cannot help believing that this proportion is somewhat below the ordinary standard; however, it being admitted that hemoptysis may prove the cause of death in some cases and may also be the first symptom of the disease, M. Louis infers that this discharge of blood might actually be at one and the same moment the *first* and *last* evidence of phthisis. We have never heard of a case of the kind actually occurring.

The author reiterates the statement made in his former edition as to the *almost* invariable dependence of hemoptysis, if at all considerable, upon tuberculization of the lungs, except in certain cases where the bleeding has followed external violence or suppression of the catamenia. Our own experience confirms the accuracy of M. Louis on this point, while it testifies to the error of Laennec, in regarding pulmonary apoplexy as the chief cause of abundant hemoptysis. Often are the anatomical characters of apoplexy of the lung obvious in the dead subject, when there has been no hemoptysis during life; in the epidemic yellow fever of Gibraltar the author ascertained that the pulmonary lesion in question was very common, while not a single individual had spit blood at the time.

The marked influence of sex on hemoptysis was exhibited in the former edition of this work; the statements there made, that of forty-two women carefully interrogated thirty-six had suffered from this symptom, while twenty-one only out of thirty-eight males had spit blood, does not, however, receive the corroboration of additional cases. It were much to be wished that the point had undergone investigation in this country, as we cannot divest ourselves of an impression that here, at least, the male sex suffers more frequently than M. Louis' figures would lead us to believe. It is curious, as mentioned by the author, and as general experience witnesses, that hemoptysis occurs with extreme rarity in phthisical *children.* The more special tendency of the malady to implicate the bronchial glands may in part account for the fact, but certainly not fully explain it; of nearly two hundred tuberculous children observed by M. Guesnard (Thèse, p. 12), two only had had hemoptysis, little girls aged nine and eleven years.

M. Louis notes the infrequency with which, comparatively speaking, any immediate cause for the escape of blood can be ascertained; our own experience upon this point coincides with the author's. Nor is the actual anatomical mechanism (if we may be allowed the expression) of the hemoptysis more readily traceable, and if before the formation of cavities we are constrained to refer it to exhalation, so too even after the establishment of ulceration must we provisionally recognize them as its common source, so singularly rare is it to detect a ruptured vessel in the interior of the lung.

The author insists upon the fact that night perspiration was not influenced, in respect of violence or persistence, by the state of other organs or tissues besides the skin; that, for example, the supervention of diarrhea, however copious, had no effect in diminishing the abundance of cutaneous exhalation. M. Louis has, in a word, seen no reason for accepting, as a general truth, the doctrine of " balance of the functions," taught by various systematic writers; the *occasional* cessation of perspiration while the intestinal flux acquires redoubled violence, he conceives himself justified in regarding as a mere coincidence. And he is desirous of impressing these results upon practitioners, as the treatment pursued by not a few persons is founded upon a very deep-rooted conviction of the reality of this fancied " balance of the functions."

The conditions of the tongue in phthisis are well known to be extremely variable; the author gives the results of comparison of its state with that of the mucous membrane of the stomach in a considerable number of cases. These results may be expressed as follows:

(*a.*) *In* 19 *cases of softening with attenuation of the mucous membrane of the stomach :*

Tongue natural in 9 cases; in the remaining 10, red at the tip or edges; in 4 of them for 15 or 20 days; in 6, for 2 or 3 days only.

(*b.*) *In* 8 *cases of inflammation limited to anterior surface of the stomach :*

Tongue pale or red an equal number of times; and in one instance the redness was quite temporary.

(*c.*) *In* 17 *cases of inflammation affecting the whole or part of the fundus :*

Tongue natural in 10 cases; in 7, slightly red at the edges, either towards the close of life, or for a few days at an earlier period.

(*d.*) *In* 19 *cases of mammillation more or less general and marked :*

Tongue of a more or less florid red colour for a variable time in 8 cases; natural in 11.

(*e.*) *In* 14 *cases of various associated lesions of the stomach :*

Tongue unnaturally red for one or several weeks in 6 cases; natural (it is to be inferred) in the rest.

(*f.*) *In* 19 *cases in which the mucous membrane was perfectly healthy in respect of colour, consistence, and thickness :*

Tongue more or less red in 10 cases; in 1 of these the redness persisted through the whole course of the disease, and was attended at one period with dryness; in the remaining 9 (it is to be inferred), natural.

These results indicate a degree of independence of the conditions of the tongue and stomach, for which the general statements of writers, especially of the Broussaisian school, ill prepare the reader; the truths they display are of deep practical importance. Another condition of the tongue occurring in one eighth of the subjects,—albuminous exudation on its surface,—is of more rare occurrence in this country than in France, as far as our own experience goes. The author has in a similar manner established its want of connexion with any particular state of the stomach. When it does occur, it is a phenomenon of singularly bad augury, as it rarely appears until a few days before death.

Of 112 patients, 5 only escaped diarrhea. In the eighth part of the cases it set in with the main disease itself, and persisted till the fatal termination, lasting thus from five to twelve months. Some individuals, whose malady lasted four or five years, laboured under almost continual diarrhea during that long space of time; in the greater number of cases, diarrhea did not supervene till the principal affection had reached the second half of its course, in some instances not till the closing days of life. The author then regards diarrhea as belonging to the last period of existence, or as of protracted existence, and inquires into the anatomical conditions attending both varieties. Ulcerations of the small or large intestine, or both, with (in four fifths of the subjects) pulpy softening of the mucous membrane of the colon, were discovered in the first class of cases,—all these lesions being evidently of recent origin, and the ulcerations small. Protracted diarrhea was either continuous or remittent.

The subjects affected with the latter form of the symptom, presented lesions not materially differing from those existing in patients whose diarrhea commenced but a few days only before their death; hence the inference, that in the present class of cases, visible lesions were but a small measure of the cause of the diarrhea,—that these lesions have too only made their appearance within the last few days of existence, and that previously to their occurrence, altered secretion was the true source of the intestinal discharge. Lastly, when the diarrhea had been protracted and continuous, and at the same time the stools numerous, vast and numerous ulcerations were discovered in the small and large intestine.

The symptoms of chronic peritonitis, it is observed by the author in a chapter making its first appearance in the present edition, are generally of moderate intensity, few in number, and often pass unperceived; nevertheless they are sufficient to announce with certainty the lesion to which they appertain. The description given of these symptoms may be condensed as follows: At a variable period of the principal affection, sometimes even at its outset, whether it be destined to destroy life in less than two months or in several years, the patient is annoyed with the first symptom of this peritonitis, enlarged size of the abdomen, or slight and sometimes general abdominal pain: both these phenomena may coexist. The pain is increased by pressure and percussion, and independent of diarrhea which is not always present, and when it is, is attended with suffering of a very different character from that of peritonitis. Subsequently fluctuation, or the evidence of gaseous distension may be detected. After having increased for a certain time, the sensation of fluctuation diminishes, and even disappears altogether, while the gaseous distension remains. In certain cases, where the accumulation of the gas occurs at the outset, without appreciable effusion, it diminishes after a time, and the abdomen becomes firmly resisting under pressure, and the outline of masses of intestine distinctly marked on the surface. Nausea and vomiting are rare, unless towards the close of life, as results of superadded *acute* peritonitis. The intensity of the symptoms may be such, that the patient's attention is wholly occupied with them; in other cases, where the organic changes are quite as advanced and extensive, the abdomen continues free from pain, and is simply enlarged and the seat of fluctuation,—the urine not being albuminous, nor the patient exhibiting symptoms of organic disease of the liver. The abdominal symptoms generally persist with a greater or less amount of intensity till the fatal termination; but cases of extremely chronic character are not wanting, in which after more or less protracted abdominal suffering, this ceases altogether, and the chest becomes the sole seat of functional disturbance.

The abdominal effusion may be very rapidly absorbed,—in seven or eight days for example; and the physician is sometimes not a little astonished to find a state of universal adhesion of the intestines through recent false membrane, in subjects who during life, had a few days before presented obvious signs of fluctuation.

In the combination of symptoms now enumerated, the author places the highest confidence, as diagnostic of peritonitis peculiar to subjects labouring under pulmonary tuberculization; through these symptoms he has been led to the diagnosis of the latter affection, in persons "who did not cough at all or did so but very slightly, and whose chest examined with extreme care, with the aid of percussion and auscultation, presented no obvious sign of disease."

Cancerous peritonitis, however, runs the same chronic course as the tuberculous variety; hence the possibility of error. M. Louis points out the less amount of fever, and the absence of sweating and diarrhea, as circumstances distinguishing the former; cancer will also commonly be found in some other organ, and the age at which this disease is usually observed differs, as is well-known, from that of subjects affected with tubercles.

The symptoms appertaining severally to ulceration of the epiglottis, larynx, and trachea, receive very close investigation at the author's hands. The particular cases related justify the general inference, that fixed pain at the upper part of or immediately above the thyroid cartilage, difficulty of deglutition and escape of drinks through the nose, announces (provided the pharynx and tonsils be perfectly healthy,) the existence of ulceration of the epiglottis; but when limited in amount, this anatomical change was often latent. Moderate pain of limited duration in the region of the larynx, coupled with more or less marked alteration of voice, signifies the existence of superficial ulceration of that organ; whereas severe and continuous pain with persistent aphonia, results from deep ulceration. But in the case of tracheal ulcerations, no matter how numerous these may be, no special symptom is commonly discoverable; in a single instance only, observed by the author, a feeling of slight heat and obstruction behind the upper part of the sternum, was noticed as the effect of tracheal ulceration,—but here the mucous membrane was most extensively destroyed. Simple inflammation of the trachea, characterized by very bright redness, with, in some instances, thickening or softening, produced sometimes more or less sharp pain, with a sensation of heat along the neck.

The statements originally made by M. Louis respecting the course and influence of pneumonia occurring in phthisical subjects, inconsistent as it has been maintained they are with general experience, are repeated with greater emphasis even in the present edition. When pneumonia makes its appearance in phthisical subjects who are still able to pursue their occupations, and whose strength and flesh have not yet diminished materially, the affection presents the ordinary series of symptoms characterizing it in previously healthy persons; but these symptoms are generally not severe, and the disease "almost always terminated by cure, even when there were tuberculous cavities at the apices of the lungs." That is, an affection of the lungs which when those organs have, like the rest of the system, been previously perfectly sound, cuts off from one third to one seventh of those attacked, becomes almost harmless when they are partially destroyed by the worst form of malady prone to affect them. M. Louis adds: "as if the cavities and tubercles, real foreign bodies in respect of those organs, were the principal existing cause of the inflammation, and necessarily, *for this reason*, diminished its peril." This argument does not appear to us either very valid or very clear. The fact has however, as we had occasion to observe in a recent volume, (Brit. and For. Med. Rev., vol. XIII. p. 382.) been corroborated by the result of M. Grisolle's inquiries on the subject. Pneumonia, on the other hand, "occurring at the close of the disease is almost necessarily fatal;" again, a proposition not very lucidly expressed. When supervening at this advanced period, and when of limited extent, it was not usually announced by any symptom; when implicating a considerable mass of lung, the ordinary symptoms, at least very closely these, are developed.

The experience of M. Louis testifies as strongly to the incurability of pleurisy occurring in the course of tuberculous disease, as to the curability of pneumonia: the former affection he believes an active agent in causing, or at least hastening the fatal event in many cases. But these propositions refer only to pleurisy with effusion,—of course, the dry pleurisy so repeatedly declaring itself during the process of the primary disease, cannot be regarded with such apprehension. Pleurisy with effusion, existing simultaneously on both sides of the chest, the author has never seen except in phthisis and in cases of gangrene of both lungs.

The introduction of a section on the state of the genital organs in both sexes affords M. Louis the opportunity of contradicting the statement, which had once obtained some vogue, that the sexual passion is increased in intensity during the course of phthisis in the male. Careful questioning has proved the very reverse to be the fact; and we have ourselves come to the same conclusion from repeated inquiries.

One female only, of all those observed by M. Louis, continued to menstruate to the last month of life. The period at which suppression occurs is subject to variation; when the disease lasted less than a year, the menstrual discharge ceased to appear on an average at about the middle period of the affection; if from one to three years, the catamenia continued until the last third. When the disease run a rapid course, the cessation of the menses appeared to coincide with the establishment of fever; when the former was of slow type, the author failed in detecting any cause either retarding or accelerating the time of alteration in the catamenia.—Upon the alleged influence of pregnancy in arresting the progress of tuberculous disease, M. Louis is unable to speak with any confidence from his own experience. He points out, however, some easy sources of error in estimating such influence; it is quite possible, for instance, that many of the symptoms of phthisis may be more obscure during pregnancy than when the uterus is empty, although the disease were advancing as rapidly as ever. Nor is it impossible that the progress of the affection might be more rapid after delivery, than at an earlier period of its course. Again, the extraordinary variety which different cases of phthisis present in respect of their progress, points to the necessity of accumulating a vast number of cases, in which the pregnancy has *appeared* to have the alleged influence, before its *reality* be admitted.

We have had occasion more than once to draw the attention of the readers of this Journal to the anatomical and other characters of that peculiar form of meningitis of children, to which the names of granular and tuberculous have been applied by its describers, Drs. Rufz, Gerhard, and P. H. Green. These inquirers appear, however, to have limited their observations to the young subject, and the thesis of M. Lediberder, with the new section introduced into the present edition of M. Louis'

volume, are the only sources from which acquaintance with the affection, as it appears in the adult, may be derived. The malady, occurring at various stages of the primary disease, commences with frontal, intense and continuous cephalagia; the face at the same time becomes alternately pale and red; the intellect grows blunted; paralytic symptoms are rare at this period; vomiting almost constantly occurs at the very outset, and its association with cephalalgia is regarded by the author as of itself strongly indicative of the presence of tubercles in the meninges. The violent headach persists for from three to twelve days, accompanied with sharp cries from time to time: the face assumes an expression of astonishment, quickly followed by one of stupor. The pupils contracted at first, become subsequently dilated. From the fourth to the sixth day delirium, commonly of a tranquil type, sometimes accompanied with agitation and increase of the general sensibility, appears. Somnolence, and eventually coma, are noticed in the intervals of freedom from delirium. When hemiplegia exists, it generally makes its appearance some days later than the cephalalgia; some considerable part of the face, or one of the eyelids only may be paralysed; and persistent contraction is witnessed instead of paralysis in certain cases.

Meanwhile the circulation and respiration undergo remarkable changes. The respiration becomes less frequent and less deep, dyspnea diminishes or disappears, except during the closing days, when on the contrary it undergoes material increase, proportional in amount to the coexisting somnolence. Even in cases where the lungs are extensively excavated, the fever disappears during the earlier periods of the meningeal affections; but it returns with intensity towards the close; irregularity of the pulse is very rare. The temperature of the skin falls and rises with the alterations of the pulse.

The duration of the disease varies between eight and fifteen days; rarely is it longer or shorter than these periods.

Three cases reported by M. Lediberder are added, confirming the details of the general description now given; the anatomical account of the meningeal disease, as we have already hinted, is not so complete as desirable, but the cases are nevertheless well deserving of deep study. If these cases show that the diagnosis of the affection may often be easily accomplished, and that it may actually be of use, (like the allied state tuberculous peritonitis,) in leading to the detection of the primary and main disease that of the lungs, there are others in which its precise nature may be involved in obscurity. A fourth case, exhibiting the strong similarity which the symptoms, both in respect of nature and catenation, may wear to those of typhoid fever, is added and will attract the attention of readers by the abundance and accuracy of its details.

Impaired power of hearing or actual deafness is occasionally observed in tuberculous subjects; and are traceable, according to M. Ménière, to the development of tubercle in the substance of the membrane of the tympanum, leading to its more or less complete destruction.

Emaciation, one of the most striking accompaniments of phthisis, set in with the commencement of the affection in one half of M. Louis' cases; in a small number of patients it commenced at the same time as diarrhea, or diminished appetite; in the third part of the cases it was first noticed along with the development of fever. The author justly insists upon the importance of the symptom, and upon its diagnostic utility in all cases, but especially in those of the latent type.

The author next turns to the history of perforation of the lung. Many of our readers are aware that, to the accurate observations of M. Louis, physicians are indebted for the earliest precise account of this occurrence, in regard of its intimate nature, its immediate symptoms, and its eventual effects. But M. Louis and his countrymen fell into the error of imagining that perforation, (with its consequence pneumo hydrothorax) was necessarily mortal, ("mortel de sa nature," Ed. 1ère, p. 488); an inaccuracy exposed by Dr. Houghton, Dr. Stokes, Dr. Barlow, and others, in this country. Indeed cases have been narrated by some of these latter observers, (vid. Brit. and For. Med. Rev., Vol. IX. p. 394,) which seem to warrant the entertainment of the idea, that perforation may be the means of prolonging life, and arresting *pro tempore* the constitutional symptoms of phthisis. For an examination of the grounds upon which this idea may be advocated, we refer to our earlier volume just quoted, and shall only say here, that M. Louis refers in the present edition to cases where life was very considerably prolonged, after the presumedly fatal occurrence, while he makes no allusion to the statements of our countrymen on the subject, with whom all the merit of original observation of the fact exclusively resides.

In illustration of the extreme variableness of the period of the primary disease, at which perforation of the pleura may occur, the author relates with much minuteness the history of a female, aged twenty-three, in whom the first evidences of phthisis had existed for fifteen days only, at the time the serous membrane underwent rupture. This, however, is an extreme instance,—one which stands alone among all the cases ob served by the author in a period of more than twenty years. Nevertheless it is a notorious fact, that although perforation does not occur in the ordinary course of things until an advanced stage of the disease, it does not very unfrequently supervene at a comparatively early period. Pleural perforation appears to be about double as frequent on the left as on the right side; of 50 cases, (by the author 10, M. Reynaud 40,) the rupture occurred in 33 on the left side, in 17 on the right.

The "march" or course of phthisis, is one of the points of its history upon which the author's labours have been most striking. He considers the subject under the three heads of acute phthisis; chronic phthisis terminating by sudden death, explicable or not by the condition of the organs; and latent phthisis. The general description of acute phthisis is founded on thirteen cases. In *three* of these, running a course of twenty, twenty-nine, and thirty-four days, the character of the symptoms was peculiar. In the midst of health these individuals were seized with rigors and more or less violent trembling, renewed on the following days. Heat followed those rigors, of a persistent kind, and as intense as in typhoid fever. From the outset, too, extreme thirst, complete anorexia, frequency of respiration, marked oppression, cough, (except in one instance, where it did not supervene till the tenth day,) and expectoration moderate in quantity were present; and whenever the patients were first observed, they were found in a state of fever.

Amid these formidable symptoms, however, there appears none clearly indicative of disease of the other great cavities beside the chest; nor were the evidences of pneumonia or pleurisy present. Hence the inference that a combination of symptoms such as the above, more especially if after a few days' duration they increase in violence in spite of treatment, and the signs of pneumonia, pleurisy, or intense capillary bronchitis are indiscoverable, indicates with much probability that a case of acute phthisis is before us. In these views M. Louis confirms the justness of the principle of diagnosis laid down by Sir James Clark, (on Pulmonary Consumption.) The author further observes, that if delirium have occurred at the outset, as in one of his cases, the chance of confounding the affection with typhoid fever is rendered possible. However, cough and dyspnea, at least of a notable kind, do not attend typhoid fever from the first, and in this affection the amount of prostration is materially greater, if the febrile action be intense, than in acute phthisis; in the former case too, the alteration of the features, and of the functions of the organs of sense is more deeply marked.

In *eight* other cases, in which death occurred from the fortieth to the eightieth day, the outset of the affection was not marked by any violent symptoms. The febrile action, as far as could be ascertained, presented at that period no great intensity; the cough was however almost always very troublesome, the appetite and strength greatly diminished; the cough nevertheless attained a marked amount of severity in one case only, and once only did hemoptysis occur at the outset. This last fact the author considers to a certain extent subversive of the commonly received notion, that hemoptysis depends on congestion of the lungs; for here are precisely a series of conditions favorable in the highest degree to that congestion, and yet observe the rarity of bloody expectoration. The hemorrhage occurring in the course of tuberculous disease he regards as "dependent upon some state of which the precise nature is undiscoverable, having for its indispensable condition the presence of tubercle." The difficulty of diagnosis in cases of this type is materially greater than in those of the former.

Eight of the author's thirteen patients had pain in the side; yet three only presented the results of recent pleurisy on post-mortem examination. The author thinks it a fair conclusion from these facts, that the pains of phthisical subjects are not always due to pleuritic inflammation; and that rapid development of tubercles may, independently of such inflammation, give rise to those pains. This idea derives support from our inability in *some* cases to detect any physical evidence of pleuritic change in the position they occupy.

The author passes seriatim in review the alterations of the various viscera in these acute cases; and shows, in the words of his conclusion, that notwithstanding the rapid course of the disease the secondary lesions were the same in nature and proportional frequency as in chronic cases, differing in truth only in respect of amount. It is remarkable that in this summary no allusion is made by the author to the aid obtainable by the physical signs in the diagnosis.

It would be doubtless a subject of congratulation could we ascertain with certainty the causes rendering the course of phthisis so rapid, as in the cases we have been considering; unfortunately the information existing on this point is far from accurate. Has *sex* any particular influence in this direction? M. Louis replies by the subjoined facts:—

| | 210 Cases. | | Mean duration. |
|---|---|---|---|
| Women . | 97 | ... | 20 months. |
| Men . | . 113 | .. | 17 ,, |

But if from these 210 cases all those which ran a course of more than a hundred months, and respecting the precise period of outset of which doubt may be very legitimately entertained, the proportions change as follows:—

| | Cases. | | Mean duration. |
|---|---|---|---|
| Women . | 94 | ... | 13 m. 28 days. |
| Men . | . 111 | ... | 14 m. 1 day. |

Hence it would appear that sex really has no special influence on the progress of the disease; and this idea is confirmed by considering in another point of view the author's facts. In truth, of 113 cases furnished by the male sex, 78 ran a course below the mean duration; and of 91 occurring among females 65 were similarly circumstanced; here the proportions are almost identically the same.

The following figures reply to the question of the influence of *age* on the duration of the disease:—

| Age. | | No. of cases. | | Mean duration. |
|---|---|---|---|---|
| 15—30 | ... | 100 | ... | 12 m. 20 days. |
| 34—45 | ... | 68 | ... | 23 m. 16 days. |
| 45—60 | ... | 26 | ... | 22 m. |
| 61—68 | ... | 11 | ... | 14 m. |

The author very justly observes that the number of cases in the last category is too small to inspire confidence in the result; and that therefore, taking the first three, we are authorized to consider youth as one of the conditions tending to give rapidity to the course of phthisis. This derives new probability from the fact that of the 13 acute cases already referred to, 11 were supplied by individuals aged between 15 and 30.

In respect of *constitution* we find these figures:—

| Constitution. | | No. of [fatal] cases. | | Mean duration. |
|---|---|---|---|---|
| Strong. | ... | 56 | ... | 14 m. 6 days. |
| Moderately strong. | | 87 | ... | 14 m. 11 ,, |
| More or less weak. | | 53 | ... | 16 m. 4 ,, |

Hence, if this comparatively moderate number of cases is to be trusted to, we must infer that weakness of constitution is rather favorable than otherwise to slow advancement of phthisis; and it seems to be therefore presumable that the same form of constitution cannot be specially effectual in its first development; though, as the author observes, this will not admit of demonstration until the proportion of persons of weak and strong constitutions in the community be ascertained. M. Louis further found that of 144 phthisical subjects, still living, when observed 45 were of strong or very strong, 72 of moderately strong, 27 of weak constitution.

*Latent phthisis.* Although the present edition contains matter simply confirmatory of the history of latent phthisis given in the first, adding nothing actually new, we cannot refrain from drawing the reader's attention to its contents. Of 123 cases of phthisis referred to by the author in his former edition, 8 (or 1-15th of the whole) were examples of the latent form of the disease—of the existence of tubercles for a period varying from six months to two years *before* their announcement by cough or any obvious pectoral symptom. In 4 of these cases there were no general symptoms of any importance for a certain period; in the 4 others, although cough, expectoration, &c. were wanting, fever, anorexia, and emaciation, &c., disclosed the existence of some serious affection. And it is truly remarkable that the fever and the general derangement of function were the most violent in three cases where all the organs but the lungs were healthy; so that the only diseased organ was the only one symptomatically free. Facts such as these bear the strongest testimony to the value of physical signs, the sole means given us of avoiding in instances of the kind the very gravest errors of diagnosis, of prognosis, and of treatment. Of the causes impressing this latent character upon pulmonary tubercle, M. Louis is unable to give any account.

The *diagnosis* of tuberculous disease next engages the author's attention. The affection is here considered divisible into two periods,—the one extending from the first deposition of the foreign matter to the establishment of softening; the other, from this latter occurrence to death or recovery, "or at least to the cessation of the principal symptoms and the more or less complete restoration of the patient to health."

During the first of these periods the fact of the cough generally supervening, without obvious cause, in a state of apparently excellent health, and its continuing dry for a variable period, may alone justify suspicion of the real nature of its cause. The sputa are at first and sometimes for a considerable time clear, frothy, mucilaginous-looking, and whitish; this is not the case in simple bronchitis. Wandering pleuritic pains in the sides, behind the clavicles, between the scapulæ, to which we have already made allusion, are in themselves almost characteristic of tuberculization; they could only be confounded with rheumatic pains complicating acute bronchitis, and this combination, especially in the absence (as admitted) of all apparent cause for the disease, is singularly rare. The statements already made respecting hemoptysis would lead the reader to anticipate the author's attaching very material importance to its occurrence as diagnostic of phthisis; its vast significance is indeed demonstrated by the fact, that of "upwards of 2400 patients carefully interrogated upon this point,—patients who had neither had an injury of the chest, catamenial suppression, nor gangrene, nor cancer of the lung,—one only had had hemoptysis of any serious amount." It would have been desirable to know in what proportion gangrene and cancer of the lung were met with as causes of hemoptysis during the period the above 2400 cases were passed in review. We should have wished also that the author had investigated comparatively the characters of tuberculous and cancerous hemoptysis. The rupture of aneurism ought to have been included among the exceptional causes of extensive hemoptysis; such rupture is not necessarily fatal. Acute pulmonary catarrh is generally preceded by coryza; the cough of phthisis sets in without any affection of the nasal mucous membrane.—Evening fever and emaciation will give further support to the inference justified by the previously-stated circumstances.

The author justly states that it belongs to physical diagnosis to decide the question of the existence of tuberculous disease; but we regret to find that percussion and auscultation alone are referred to. The statements respecting the results of percussion present no novelty; nor are the more delicate points of inquiry in this matter made the subject of examination. It is not enough to consider the "sonorousness" of the part percussed, or to speak of diminution of this sonorousness as announcing consolidation; the special character of the tone elicited requires quite as much—often more—close analysis; and signifies more precisely the state of the lung than the mere diminution in clearness or loudness ever can.

In the majority of cases the "character of the respiratory murmur undergoes appreciable changes, even before the sonorousness of the chest is affected;" M. Louis then agrees with close observers in general, that auscultation is available as a means of diagnosis at an earlier period than percussion. He is of opinion that in estimating the value of harsh, bronchial respiration, as a sign of disease at the summit, it is absolutely necessary to take into consideration the side at which it presents itself, as harshness of character and prolongation of respiration are more common under the right than the left clavicle as a natural condition. The author points out, too, the *naturally* more intense resonance of the voice at the right than the left summit as a condition which requires to be carefully borne in mind in estimating the signification of bronchophony.

The author regards "dry and humid crackling" occurring at the summit (and which may set in before even distinct bronchophony is established) as "the indication of a certain quantity of mucus, which may be secreted long before the softening of the tubercles present, nay, while semi-transparent granulations constitute even the whole existing tuberculous change." This is a point of very serious importance.

The author next enumerates, with rapid comment on each, the several secondary lesions to which reference has already been made, and which afford more or less positive indirect evidence of the existence of tuberculization of the lungs. We mean double pleurisy, ulceration of the larynx, chronic peritonitis, and the special form of meningitis above briefly described. Diarrhea of long continuance, accompanied with emaciation, and obstinately resisting all modes of treatment, M. Louis regards as almost exclusively the apanage of phthisis, and capable of leading to the diagnosis of that disease, in cases where neither cough, expectoration, hemoptysis, nor pain in the side existed.

The pages devoted to the diagnosis of the second period of the disease, present little which is not sufficiently well known, to absolve us from the necessity of analysing their contents. In commenting upon the cracked-metal character of the percussion-note furnished by large cavities, the author states that he has, in cases of pleuritic effusion and simple pneumonia of the upper lobe, detected the modification of sound. That the *tubular or amphoric note* (such as is produced by sharply filliping the cheek while the mouth is fully but not very forcibly distended with air) is sometimes to be elicited in the class of cases referred to by M. Louis, is matter of established doctrine in this country; and we believe that it is this peculiar note which is meant by him and not the perfect *cracked-metal character.* At least, we have never encountered this modification of sound under the circumstances supposed, and its very mechanism in the cases in which it really is producible points to the extreme improbability, if not actual impossibility, of its being generated in cases of simple pleurisy and pneumonia.

Phthisis terminates almost invariably by death, says M. Louis, whether this occurs a few weeks or several years after the outset of the disease. In some rare cases, however, he admits that a favorable issue occurs, and that after having suffered from the severest symptoms the patient is able to return to his ordinary occupations. Three cases are here briefly sketched, in which the disease appears distinctly to have undergone suspension of progress. The author well observes that such cases are in all probability of not very unfrequent occurrence, but that they often pass unnoticed. The statements here given add nothing to our knowledge on the curability of phthisis; it is not the occasional cessation of symptoms of the disease and apparent restoration to health which require to be demonstrated, (all are aware that such fortunate changes do sometimes occur,) but the proportion of cases in which such changes may be expected, and above all the circumstance of age, sex, climate, habitation, constitution, occupation, &c., which may exercise appreciable influence on their accomplishment. Connected, of course, with the latter system of inquiry is the investigation of the therapeutical means best calculated to facilitate the natural processes by which the suspension of the malady is effected.

The researches of the late M. Rogée lead to the inference that anatomical cure of tuberculous disease is a phenomena of much more common occurrence than we should upon any other evidence be justified in believing. Cretaceous and calcareous deposition in the lungs, he, in every instance, regards as the result of transformation of tuberculous matter, and such deposition he discovered in the lungs of fifty-one out of one hundred old women dying at the Salpêtrière, into which institution admittance is not granted until the applicant shall have attained her sixtieth year.

Upon the cases of the three patients just referred to, M. Louis remarks, —"it is worthy of note, that these three persons had passed their fortieth year; that only one lung had been, or appeared to have been, tuberculous; that in two of the cases the cavities had to all appearance retained their primitive dimensions; that the patients belonged to different classes of society; that the outset of the affection was marked by symptoms of much severity in one case only, and that in none of the three could the favorable course of events be ascribed to the influence of treatment." To these observations we may add that in the three cases the *right* lung was the organ affected; and that in the immediate neighbourhood of the single cavity, lined with false membrane existing in one of these cases, were two tubercles of the size of a very small nut in an incipient state of softening: the former fact lends support to the observations already made (p. 427) regarding the questionable accuracy of M. Louis' inference as to the greater frequency of implication of the left lung.

Thirty-six pages are devoted by the author to an examination of the *causes*—predisposing and exciting—of tuberculous disease. The general inference from these is depressing to the last degree; for it is none other than that the most profound ignorance prevails on the subject. We knew it before we saw these pages; we know it more fully and thoroughly now. Still this section of the volume is far from being without its value, for it furnishes a philosophical exposure of the fallacies into which authors, prone to find *causes* in *antecedents*, necessarily run. A few examples will suffice. *Weakness of constitution* is set down as a cause of phthisis, and the influence of such constitution appears so readily traceable in the development of the disease that it has been universally admitted. But where is the demonstration of the alleged fact? Who has ascertained the proportion of weak and strong constitutions in a given population, and ascertained the ratio in which each class furnishes the victims of phthisis? Yet until this be done, it is obvious that no shadow of importance can be attached to even the most speciously attractive reasoning in favour of the destructive agency of weak constitutions. The more so, as we have seen that M. Louis found the disease run a somewhat more rapid course in subjects of strong than feeble constitution.

The reality of hereditary influence in the production of phthisis is so universally admitted that it would seem a sort of scientific heresy to doubt it. Yet, it may be asked, how has it ever been *proved*? What demonstration has ever been given better than the often reiterated statement (the truth of which is indubitable) that persons often die phthisical whose mothers or fathers were so before them. But obviously, in the instance of a disease so common as phthisis, this affords no sort of demonstration. In order to ascertain the reality and the amount of the influence in question with surety, it would, as the author observes, be necessary to have "tables of mortality, by means of which an equal number of subjects, born of phthisical parents and of persons whose fathers and mothers were not tuberculous, might be compared together." A Parisian hospital physician, M. Briquet, has rather recently published some statistical results respecting phthisis to which undue importance is very likely to be attached; one of these results appertains to the present matter, and we subjoin it:

| Phthisical subjects. | Parents healthy or non-tuberculous. | Parents phthisical. | Parent's health unascertainable |
|---|---|---|---|
| 67 Males ... | 37 ... | 24 | 6 |
| 32 Females ... | 14 ... | 12 | 5* |

Hence it would follow that, taking both sexes together, about one third of phthisical subjects spring from parents similarly diseased. But, "if the mortality from phthisis at the Necker Hospital (of which M. Briquet is physician) were during three years $\frac{11}{37}$ of the whole, or a little less than one third, and if this ratio were general through the capital, it would signify that $\frac{11}{37}$ of the population of Paris die phthisical, and that, consequently, whenever engaged in the study of hereditary influence in connexion with any disease, we must expect to find tuberculous parents eleven out of every thirty-seven times; so that if the proportion were found the same in the parents of tuberculous subjects, it would follow that hereditary transmission is really without influence in the case of phthisis." It is to be observed that all these *ifs* are attached to propositions which are in all probability positively ascertainable.

The alleged influence of the use of stays on the development of

* There is some slight error here in the figures in the original.

phthisis is possibly a matter of mere assertion, according to the author. He might have gone further and affirmed it to be actually so. We know of no demonstration in any language on the subject, and are indeed unacquainted with any serious attempt to prove the reality of the baneful influence which it is the fashion to dwell upon. Many of the females submitted to the author's observation had, it is true, laboured under difficulty of breathing for a long period before they became phthisical; but the number of men similarly affected was not less considerable. And besides the majority of the author's patients had been brought up in the country, and not worn stays until their removal to Paris, at an age when they had ceased to grow, and when consequently the action of stays could have little or no influence on the dimensions of the thorax. The question in England is somewhat different, for with us stays are much more constantly worn at all periods of the day,—the desire to produce smallness of waist is also perhaps stronger and more prevalent here than in France, in proportion as the width of pelvis is less among our women than the French; for it is not so much positively small measurement that is coveted, as smallness in comparison with the dimensions of the figure on the level of the hips. But it is obvious that until we have the opportunity of comparing two series of women in all other respects similar, except that the one shall use stays at an early period of life, employ them constantly, and keep them very tightly laced, and the other not use this artificial support, or use it but to a very limited extent, we shall continue without the means of deciding whether and to what amount the alleged influence be real or not.

III. Treatment. We must refer the reader to the work itself for much detail respecting many of the other alleged causes of phthisis; our remaining space we feel called on to devote to the consideration of the author's Third Part, in which the treatment of the disease is made the subject of consideration. A chapter of this part contains the results of M. Louis' inquiries into the real efficacy of the various medicines which have more or less recently been brought into notice as capable of arresting the course of phthisis or curing it altogether.

The *protiodide of iron* stands first on the list. This medicine, the introduction of which into medical practice M. Louis erroneously ascribes to M. Dupasquier of Lyons,* was, we believe, first employed by this person in the treatment of phthisis. The flattering accounts published by him so far back as 1835, appear not to have excited any general attention, until within the last two years, a fact not a little surprising when we consider that these accounts embrace such statements as the following, "the protiodide of iron is borne well by phthisical patients to the amount of from twelve, to thirty or forty drops daily," [how the solution is prepared, and in what proportion it contains the solid salt, we are not told,] "and exercises its influence especially on the lung. Its effects generally become manifest in the space of eight days; out of every *ten patients labouring under the disease in the third stage, at least six or seven derive notable relief from its use.* At the end of a few days prompt diminution, amounting almost to suppression, of expectoration; relief of cough and

* It is sufficiently notorious that Dr. A. T. Thompson used the salt in question medicinally at an earlier period than this.

dyspnea; diminution and eventually suppression of the sweats; diminished rapidity of the pulse; decreased heat and fever; increased strength and appetite are noticed. . . . . . . . In some cases in spite of the improvement of the symptoms, the patient grows weaker and weaker, and gradually perishes; but frequently on the other hand, and in cases where the presence of a cavity had been satisfactorily ascertained, the amelioration becomes from day to day more evident, the patient recovers flesh, the cough and fever disappear, the patient regains his gaiety, and leaves the hospital in a state of cure which it is permitted to hope may sometimes be definitive." M. Louis, encouraged by these most striking announcements, and informed in personal interviews with M. Dupasquier, of the manner of preparing and administering the medicine, employed it in upwards of sixty cases occurring in either his hospital or private practice, and "to his astonishment ...... *in not a single case did he observe any amelioration which could be attributed to the new agent.*" Still, on receiving the positive assertions of M. Dupasquier, and believing it difficult to admit that this physician was mistaken, "at least very frequently" in his diagnosis of phthisis, M. Louis thinks it worth while recommending a further trial of the medicine. We have ourselves used Squire's solution of the salt pretty extensively, but as yet without forming an opinion sufficiently definite, for we have not yet analysed our cases, to justify us in laying it before the reader.

*Common salt* has of late been forced on the attention of the profession by M. A. Latour, as capable of arresting, nay, of curing advanced phthisis. Here is one of his "cases." "Mademoiselle B. aged 14; phthisical to a very advanced amount, cavern at the apex of the right lung, extreme emaciation, general symptoms extremely severe. This patient was seen by Drs. Scott and Baron. The treatment commenced the 13th April, 1839, was terminated by the end of May, and *followed by complete cure.*" Assuredly statements such as this savour more of the charlatanism of Mr. Morrison, than of science; but knowing, it appears, that M. Latour was a conscientious person, our author gave salt, during five successive months, to all the patients received into his wards at the Hôtel-Dieu. "In no single case, however, did he observe any appreciable effect produced in the state of the functions. Some patients could not go on with the chloride for more than a few days, the greater number took it for a month and upwards."......

*Subcarbonate of potass* has been recently recommended as a general solvent for all kinds of swellings and engorgements, and as exercising its powers specially in respect of tubercle. But as from his statements, M. Pascal, the author of the recommendation in question, appears like most vaunters of new drugs, to be utterly deficient in the power of distinguishing one disease from another, (judging at least from his printed details,) M. Louis considered himself conscientiously absolved from the necessity of administering subcarbonate of potass.

Dr. Cless, of Stuttgard, derives astonishing advantage (as he says) from the administration of *sal ammoniac* in large doses. The *chloride of lime* is quite as effectual in the hands of M. Hirzog, of Posen, who demands our conviction of his accuracy on the faith of such admirably narrated cases as the following:—"A man, aged 28, presented all the symptoms of phthisis, voided puriform sputa, and left the hospital

*cured, after a treatment of fourteen days.*" The excellent M. Hirzog probably has just as lucid notions about what constitutes phthisis, as it appears he possesses upon the kind of evidence which will now-a-days convince rational beings. But the truth is, a great number of the Germans are in the happiest of all possible conditions for "curing phthisis" readily,—in perfect ignorance of the principles of physical diagnosis, they trust to the local and general symptoms for their guidance, and their acquaintance even with these is superficial and routine-like,—how often chronic bronchitis, simple chronic induration, chronic pleurisy, &c., must be confounded with phthisis under such circumstances is sufficiently obvious. There are of course numerous exceptions to this sweeping statement, but we affirm that in the smaller towns, it will be found to give a fair notion of the proficiency of the medical inhabitants in respect of pulmonary diagnosis.

The cases published some years since by M. Cottereau, in illustration of the curative influence in phthisis of *inhalation of chlorine* are next submitted to analysis by M. Louis. And it clearly appears from this analysis, that not a single one of the cases in question proves the real efficacy of the alleged specific. Some of them are deficient in detail to such a degree, that it is impossible to determine the nature of the disease treated; others are examples of pneumonia or pleurisy occurring in subjects with tuberculous lungs, and it is obvious that with the removal of the pneumonia or pleurisy (which be it observed was in all probability rather retarded than accelerated by the chlorine inhalation,) great improvement in the symptoms must have taken place, and naturally been ascribed to the influence of chlorine on the tuberculous disease, inasmuch as the complications of this had been overlooked. The author, notwithstanding the unfavorable issue of the scrutiny, submitted upwards of fifty phthisical subjects to the action of chlorine, and "without in a single case obtaining a successful result." General experience on this matter supports completely the unfavorable estimate of M. Louis.

The virtues of *digitalis* are next submitted to examination. England having produced the vaunters of this drug, our readers are doubtless acquainted with the wonderful cures ascribed to its agency, and not less familiar with the fact, that no one at the present day has the shadow of confidence in its alleged specific influence on phthisis.

An Italian practitioner, named Fantonetti, has cured *cavities* by the administration of prussic acid *in seventy-two days!* This is quite enough on the subject of Signor Fantonetti and prussic acid.

Creosote has had its supporters as a healer of tuberculous cavities; but no evidence exists in its favour of a kind to claim the consideration of rational persons.

The author closes his list of reputed specifics with iodine, which he does not appear to have tried upon a large scale himself.

Our first inclination was to regret that M. Louis had not experimented with emetics, which have perhaps been more cried up than any system of medication of late years. But perhaps, on considerations of humanity, we have reason to rejoice that some hundred individuals additional were not submitted to the misery of vomiting every second morning for some weeks or months; for we confess that what we have heard or read on the powers of these medicines has left no other impression on our minds than that of the facility of belief—if it would be discourteous to use the word credulity—of those who administered them. M. Louis might have ascertained the names of several other specifics for phthisis from the works of young gentlemen in this country, laudably desirous of notoriety and practice; but as these names have escaped him we shall allow him to remain in his ignorance,—it would be hardly fair, indeed, to spread these discoveries abroad, and so run the risk of filching from the original observers the reward of their patient zeal and most amiable disinterestedness.

But if the brief survey now given demontrates the utter insignificance of all declared specifics, it by no means justifies us in supine carelessness as to the discovery of a medicine endowed with the special attribute of modifying the course of phthisis. The efforts of those who are placed in a position fitted for the purpose should be unceasing in the search after such a medicine; for nothing can be more unphilosophical than to conclude than it does not exist, because it has not yet been found. It is manifestly not from the οι πολλοι of drug-lauders that the discovery of so inestimable a boon is legitimately to be expected,—not from those who seize with avidity upon each new medicine introduced into the market and administer it pell-mell in every form of human ailment,—invariably, according to their own accounts, with the "happiest effects:"—it is from him who, thoroughly versed in the diagnosis of disease, has enough of incredulity in his intellectual composition to doubt the evidence which is not repeated time after time in similar cases,—who has a fund of patience which no labour can exhaust, and a conviction of the grandeur of his task which disappointment, be it repeated ever so often, can never succeed in shaking. And if the general truth, that men of this noble stamp are rare, give us just cause for apprehension that the day of success is yet far distant, we have still fair motive for high hope in the fact that no profession perhaps numbers so many thus endowed in its ranks as the medical.

The author turns to the subject of rational treatment, commencing with a lucid and extremely practical chapter on the means best calculated to ward off the development of the malady in those constitutionally predisposed to it. We shall not extract from this chapter however; because, admirable as it is, the subject has been so completely exhausted by Sir James Clark, that nothing actually important and at the same time novel to the reader of the works of our countryman, could be easily discovered. It is most gratifying to find that the first pathologist of continental Europe has given the weight of his influence to the study of these purely practical matters; especially as it is impossible but that his example will be followed by many of his disciples.

We shall dwell somewhat more at length on the author's views respecting the treatment of the actually formed disease. During the earliest period of the chronic malady, at a time when the physical signs united to the local and general symptoms scarcely suffice to furnish an exact idea of the patient's real state, if there be no particular fever, diarrhea, nor severe thoracic pain, and if the patient be of lymphatic temperament, slightly tonic vegetable infusions should be prescribed; and if the cough be troublesome, some preparation of opium or stramonium in addition. Inhalation of aqueous or narcotic vapours should be had recourse to several times daily, if the cough resist the latter medicines taken by the mouth, or the former used in enema. The recommendation of tonic drinks at the outset of phthisis must be so novel, to the French reader especially, that we are not surprised to find the author entering into an explanation of the motives which guide him in authorizing it. His arguments may be briefly stated thus: the tuberculous affection is a *general* disease of the system, and hence local remedies must be insufficient to check it, a general treatment being the only one likely to modify the constitution; for persons of the stamp above referred to, tonic medicines are the best fitted, the exhibition of mucilages and the observance of a milk diet are, as far as knowledge goes of the probable causes of tuberculization, means calculated rather to increase than diminish the evil. M. Louis never advises the use of asses' milk, except in those very rare cases of excessive sensibility of the stomach, in which that organ will not bear any other sort of nourishment. In large towns he is strongly opposed to its employment; believing that in these the asses must, as well as the cows, frequently themselves grow tuberculous.

To the vegetable bitter the author would recommend the addition of some ferruginous water at meals (Bussang, Spa, Pougues, &c.,) or some artificial preparation of iron, such as the protiodide. Evening thirst should be relieved by diluents having the same temperature as the apartment, or by a little very very weak chicken-broth.

M. Louis expresses himself most emphatically against the use of issues;—"neither in hospital nor private practice have I in a single instance seen any amelioration produced which could be legitimately ascribed to them."

The importance of avoiding exposure to sudden alterations of temperature, &c., is duly considered; and in connexion with this, the propriety of sending phthisical patients to winter in a warmer climate than their own examined into. Like Sir James Clark, the author counsels removal to warmer latitudes in the very earliest stages of the disease, and on the same grounds as our experienced countryman. But, like him, he admits that a successful result is by no means to be predicated with certainty; and states that he has known females pass two successive winters in warm climates, return to Paris and go through the succeeding winter more favorably there than in their more southern place of sojourn. The author has less confidence in the utility of sea-voyages than many of his predecessors.

Upon the absurdity of submitting these patients to a severe system of diet the author well insists: their food should be succulent, and insuring the greatest amount of nutritive property in the smallest space.

If, contrary to what has so far been supposed to be the case, there be much fever present, until this be removed it is the author's opinion the tonic preparations should be given up and mucilaginous drinks substituted: we believe that in the great majority of cases this will be found correct practice, but are persuaded that instances occur in which ferruginous preparations may be continued with advantage, even during the existence of feverish action of some intensity.

But persistence in the system of treatment now indicated may be rendered impossible, for a time at least, by the occurrence of some one or other of the urgent symptoms to the appearance of which the phthisical subject is almost from the first hour of his illness more or less exposed.

The author considers with much minuteness the various modes of combating these, (hemoptysis, local pain, sweating, &c.;) but we do not discover any suggestions here likely to be new to those acquainted with the writings of English authors. Nor shall we analyze the pages devoted to the consideration of the disease in its second stage; whatever be the wisdom of the therapeutical views here taken—and it is remarkable—we find in them no actual originality, no motive for believing that the author possesses the means of prolonging the life of the consumptive beyond the term attainable by his brethren.

Our task would have been terminated, were it not that some interesting remarks on the probable means of acquiring more deep knowledge of the causes and therapeutical management of the tuberculous affection, present themselves in the preface, and imperatively call for notice. The author is persuaded that success of any consequence in these directions can only be obtained by the united efforts of a large body of physicians placed in various circumstances,—some of them attached to large public establishments, others engaged in private practice only, medical officers in the army and in the navy, &c. But what are the qualifications required on the part of the observer to render him fit to take part in the great work,—what the conditions under which those qualifications should be employed? The first element of capability on the part of the physician is the possession of intimate acquaintance with the natural course of the disease. Infinitely true; but unfortunately this truth has the effect, *in limine*, of cutting down the number of capables most wofully: does one of every hundred practitioners, taken *en masse*, understand the "natural course of the disease;" is he familiar with its diagnosis? We fear not. To medical practitioners thus qualified, however, M. Louis would assign the task of keeping full and accurate histories of all their private patients, comprising details upon the various places of habitation of these persons (rather a difficult task for those practising in these days of railways and steam,) their education and bringing up, their mode of life at all periods of their existence, the affections to which they have been subject or have suffered under, their constitutional state, the mode of progress of the malady, &c. &c. If the individual belong to the working classes, all the circumstances attending the trade he follows, the age at which he commenced to work at it, the number of hours daily devoted to its labours, &c., should be clearly made out. Besides, the health of parents and of all other near relatives should be ascertained, &c. If researches of this kind were continued for a certain number of years by a sufficient number of persons in cities and in country districts, in inland situations and on the sea-shore, in mountainous and in low localities,—applied to soldiers and to seamen, and, in a word, to individuals placed in all possible varieties of hygienic condition;—*if* all this were done, doubtless the world would be much more deeply conversant with the etiology of phthisis than it now is. But when shall we find the enthusiastic *corps* ready to take upon itself the Herculean task which the ardour of the great master has thus traced out? Upon this head we are doomed to conjecture.

Further, M. Louis, in the hope of elucidating the real influence of climate, proposes the institution of a band of travelling physicians, whose duty it should be to study the laws regulating in different latitudes the progress of the more severe classes of disease. We do not see why the resident physicians may not be deemed, from their long experience, more capable of throwing light on such points than those who (however much superior in respect of pathological acquirement) pass but a few fleeting hours on the spot; more especially, as M. Louis admits, that the wanderer must depend in great measure for his information upon the stationary practitioner. And again, we believe the experience of those countries or universities which do actually supply the funds for travelling observers, deposes in faintest accents indeed as to the utility of such persons. M. Louis, wrapped in his scientific aspirations, forgets the temptations to which the young are exposed in many of those countries to which their mission would invite them,—he dreams not of the enervating influence of those climes where the *dolce far niente* is the summum bonum of existence, and where materialities of all the most intoxicating kinds conspire but too triumphantly to drive philosophy from the field.

But whatever may be the estimate formed of the actual feasibility of M. Louis' scheme, none can fail to be impressed with deep feelings of respect for its projector. Had an individual of less elevated moral attributes laboured so successfully as *he* has done in unravelling the difficult windings of phthisical disease, the successful investigator would have been disposed to exert all his art to prove, if not that the goal was reached, at least that it was unattainable,—that further improvement was an impossibility. M. Louis here exhibits his intellect and his character in a new light; one which must contribute to shed still brighter lustre on a name, than which a more illustrious graces not the Court of Science.

---

